# Mmp2 Deficiency Leads to Defective Parturition and High Dystocia Rates in Mice

**DOI:** 10.3390/ijms242316822

**Published:** 2023-11-27

**Authors:** Rotem Kalev-Altman, Gal Becker, Tamar Levy, Svetlana Penn, Nahum Y. Shpigel, Efrat Monsonego-Ornan, Dalit Sela-Donenfeld

**Affiliations:** 1 The Koret School of Veterinary Medicine, The RH Smith Faculty of Agriculture, Food and Environment, The Hebrew University of Jerusalem, Rehovot 7610001, Israelnahum.shpigel@mail.huji.ac.il (N.Y.S.); 2 The Institute of Biochemistry, Food Science and Nutrition, The RH Smith Faculty of Agriculture, Food and Environment, The Hebrew University of Jerusalem, Rehovot 7610001, Israelefrat.mo@mail.huji.ac.il (E.M.-O.)

**Keywords:** MMP2, MMP9, matrix metalloproteinase, gelatinase, double knockout, dystocia, fibrosis, myometrium, uterine

## Abstract

Parturition is the final and essential step for mammalian reproduction. While the uterus is quiescent during pregnancy, fundamental changes arise in the myometrial contractility, inducing fetal expulsion. Extracellular matrix (ECM) remodeling is fundamental for these events. The gelatinases subgroup of matrix metalloproteinases (MMPs), MMP2 and MMP9, participate in uterine ECM remodeling throughout pregnancy and parturition. However, their loss-of-function effect is unknown. Here, we determined the result of eliminating *Mmp2* and/or *Mmp9* on parturition in vivo, using single- and double-knockout (dKO) mice. The dystocia rates were measured in each genotype, and uterine tissue was collected from nulliparous synchronized females at the ages of 2, 4, 9 and 12 months. Very high percentages of dystocia (40–55%) were found in the *Mmp2*^−/−^ and dKO females, contrary to the *Mmp9*^−/−^ and wild-type females. The histological analysis of the uterus and cervix revealed that *Mmp2*^−/−^ tissues undergo marked structural alterations, including highly enlarged myometrial, endometrial and luminal cavity. Increased collagen deposition was also demonstrated, suggesting a mechanism of extensive fibrosis in the *Mmp2*^−/−^ myometrium, which may result in dystocia. Overall, this study describes a new role for MMP2 in myometrium remodeling during mammalian parturition process, highlighting a novel cause for dystocia due to a loss in MMP2 activity in the uterine tissue.

## 1. Introduction

Parturition is the final and most critical step for successful mammalian reproduction. During pregnancy, the uterus undergoes a period of quiescence, which is fundamental for fetal growth and development. However, at some point, dramatic changes occur in myometrial contractility, resulting in the efficient expulsion of the fetus. These events are necessary for the survival of both the fetus and the mother. However, their underlying mechanisms are only partially defined. Additionally, since preterm births account for ~11.5% of all live births in the US and are the main cause of perinatal mortality and morbidity worldwide [1,2], elucidating the molecular pathways that control parturition will contribute to combating preterm births or, on the other hand, to optimizing protocols for medically induced labor [3].

In all mammals, the uterine wall consists of three major elements: (1) the endometrium; (2) the myometrium, which consists of an inner circular layer and an outer longitudinal layer of oriented smooth muscle; and (3) the perimetrium outer layer. Notably, the endometrium, which contains multiple secreting glands, undergoes extensive remodeling throughout the estrus cycle [4]. Moreover, the myometrium layer of the uterus undergoes a transition from a quiescence state during gestation to contractility mode during labor, a mandatory shift for executing natural parturition. By secreting and/or responding to progesterone and estrogen signaling, the myometrium plays an essential role in regulating its activities, which also involves profound tissue remodeling, which, in turn, regulates the myometrium’s activities. However, while research from past decades has provided critical insights into the parturition process, the molecular mechanisms involved in its initiation remain largely unknown [1,2,5,6].

Matrix metalloproteinases (MMPs) are a large family of enzymes known for their ability to degrade different components and proteins in the extracellular matrix (ECM). Therefore, they were found to be involved in many physiological processes, such as in ovulation and embryo implantation [7,8,9,10]. For example, Novaro et al., showed that in rats, at the time of implantation, the blastocyst starts to produce nitric oxide, which leads to increased levels of MMP2, which is necessary for the tissue-remodeling process that takes place in the implantation sites [7]. Such MMPs were also found to participate in pathological conditions, including cancer-cell metastasis, multiple sclerosis and Alzheimer’s disease [11,12,13,14]. The gelatinase subfamily of MMPs has only two members, MMP2 and MMP9. These secreted enzymes share a similar protein structure, comparable biological activity in different biological systems and shared substrates in vitro, such as gelatin [15,16,17,18,19]. In addition, we previously found that during development, both MMP2 and MMP9 are required for executing the migration of the unique population of neural crest cells (NCCs) in both chick and mouse embryos, and each MMP compensates for the loss of the other [20,21,22]. On the other hand, in our recent study on skeleton development, we found that each gelatinase has an individual and unique role; while MMP2 regulates intramembranous ossification affecting the development of the skull and the cortices, MMP9 controls the endochondral ossification affecting the longitudinal growth of the skeleton, as well as the overall body length [23]. Hence, their redundant or individual activities are highly context-dependent.

Interestingly, several MMPs, including MMP1, MMP2, MMP3, MMP7, MMP9, MMP10 and MMP11, and TIMPs such as TIMP1, TIMP2 and TIMP3, were previously found to be expressed in the uterus in various species [24]. Furthermore, their balanced function was shown to be essential for normal uterine tissue remodeling throughout the estrus cycle, as well as during pregnancy, parturition and postpartum uterine involution in both rodents and humans [24,25]. For example, Engelen et al., showed that in cows, there is an increase in MMP2 levels, right before calving, which is associated with collagen degradation that allows cervix ripening [26]. Furthermore, Vadillo-Ortega et al. described how in humans, MMP9 activity increases in amniochorion with the onset of labor [27]. These studies suggested that MMPs and TIMPs participate in the dynamic regulation of uterus remodeling. However, the comparable or individual roles of each MMP have not been explored. Here, we set out to determine whether the gelatinases, MMP2 and MMP9, have a combined or individual role in uterine function during parturition process, using single- and double-knockout (dKO) mice for both. 

## 2. Results

### 2.1. Mmp2 Loss Results in Defective Parturition Process and Dystocia

**Figure 1:**
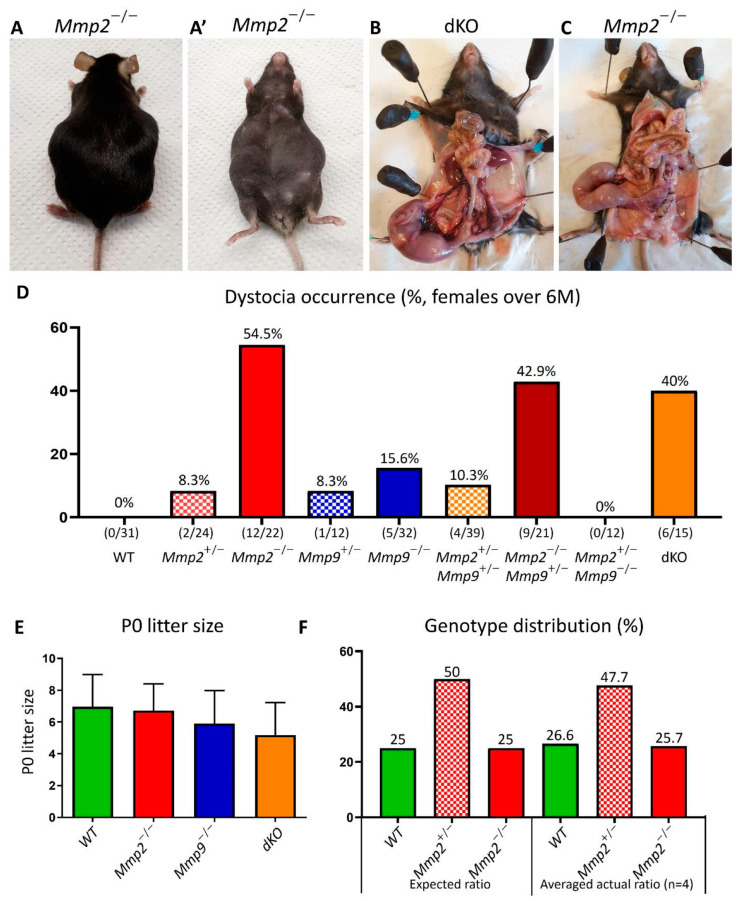
High percentage of dystocia in *Mmp2*^−/−^, *Mmp2*^−/−^*Mmp9*^+/−^ and dKO females. (**A,A’**) Dorsal and ventral view of *Mmp2*^−/−^ female that underwent dystocia at the end of the pregnancy. (**B**,**C**) When abdomen was opened, one or more fetuses were found dead in the uterus. (**D**) Dystocia percentage according to *Mmp2* and/or *Mmp9*-KO genotypes demonstrating its higher occurrence in *Mmp2*^−/−^, *Mmp2*^−/−^*Mmp9*^+/−^ and dKO females (red, claret and orange columns, respectively), compared to the other genotypes. (**E**) Litter-size measurements at P0 show no difference in *Mmp2* and/or *Mmp9* KO litter size compared to WT; *n* = 37 for WT or *n* = 11 for each KO (the result presented in panel (**E**) was published in [23]). (**F**) Genotype distribution from mating *Mmp2*^+/−^ female and male show no difference in generation of *Mmp2*^−/−^ embryos (*n* = 4 litters).

Dystocia is defined as a difficult and prolonged process of delivery with a negative outcome. When breeding the different MMP2/MMP9-null colonies, we frequently observed pregnant females with dystocia. Hence, we decided to methodologically characterize the occurrence of dystocia in the following genotypes: WT, *Mmp2*^+/−^, *Mmp2*^−/−^, *Mmp9*^+/−^, *Mmp9*^−/−^, *Mmp2*^+/−^*Mmp9*^+/−^, *Mmp2*^−/−^*Mmp9*^+/−^, *Mmp2*^+/−^*Mmp9*^−/−^ and *Mmp2*^−/−^*Mmp9*^−/−^ (dKO). Dystocia was diagnosed in both timed and untimed pregnancies; in the timed pregnancies, dystocia was determined when a pregnancy reached gestational day (GD) 22.5 according to vaginal plug observation, but did not result in normal parturition, as reflected by the females that which still had one or more (dead) fetuses in their uterus. Other timed pregnancies that resulted in vaginal bleeding at GD18.5–GD21.5 without any live newborns during the following 24 h, but with dead fetuses remaining in the uterus, were also diagnosed as dystocia. Additionally, untimed pregnancies in which the females had reached the end of pregnancy without any apparent parturition or newborns in the cage, but with dead fetuses remaining in the uterus, were also considered as dystocia (Figure 1A–C). In all cases, the females were euthanized.

The dystocia percentages were calculated for the females under and over 6 months (M) of age and were measured for each female’s-genotype as the number of pregnancies ending in dystocia versus the total number of pregnancies for which the female had the same genotype. Markedly, when the females were older than 6 M, 54.5% of all the *Mmp2*^−/−^ pregnancies (12/22) resulted in dystocia (Figure 1D). In contrast, no cases of dystocia were found in the WT females (0/31), and 15.6% of the *Mmp9*^−/−^ pregnancies (5/32) resulted in dystocia (Figure 1D). Additionally, both heterozygous *Mmp2*^+/−^ and *Mmp9*^+/−^ showed a dystocia rate of 8.3% (2/24 and 1/12, respectively) in the >6 M-old females (Figure 1D). Moreover, measuring the dystocia frequency among females with double mutations in the *Mmp2* and *Mmp9* genes, such as the mixed genotypes *Mmp2*^+/−^*Mmp9*^+/−^, *Mmp2*^−/−^*Mmp9*^+/−^, *Mmp2*^+/−^*Mmp9*^−/−^ or double-full-knockout (*Mmp2*^−/−^*Mmp9*^−/−^, dKO), revealed varied results, such as 10.3% (4/39), 42.9% (9/21), 0% (0/11) and 40% (6/15), respectively (Figure 1D). Notably, in the females younger than 6 M, we found much lower rates of dystocia in general, but the pattern of higher dystocia occurrence in the *Mmp2*^−/−^, *Mmp2*^−/−^*Mmp9*^+/−^ and dKO, compared to either the WT or the other *Mmp9* mixed genotypes, was similar (Figure S1A). This ruled out the possibility of these high percentages being age-related. Intriguingly, these data show that the loss of one or two alleles of *Mmp9* to the *Mmp2*-KO background (i.e., *Mmp2*^−/−^*Mmp9*^+/−^ and dKO) leads to a somewhat reversed phenotype of a decreased dystocia incidence (40-42.9%) in relation to the rate in the *Mmp2*^−/−^ females (54.5%) (Figure 1D). Overall, this analysis suggests that the occurrence of dystocia is largely linked to *Mmp2* loss, as well as indicating that the additional loss of the other gelatinase, *Mmp9*, reduces this phenotype to a certain extent.

Next, we evaluated whether the failure to undergo normal parturition is linked to in utero fetal death. The viability of the fetuses and litter size were measured at GD18.5 and P0, revealing that regardless of the genotype, the entire litter was alive at GD18.5, with a similar number of fetuses at P0 (5.18–6.97) (Figure 1E). Notably, when dKO pairs were mated, a smaller litter size was found compared to when either WT/*Mmp2*^−/−^ or *Mmp9*^−/−^ parents were mated, but this was not statistically significant (*p* = 0.0508, Figure 1E). Furthermore, the measurement of the genotype distribution of the WT, *Mmp2*^+/−^ and *Mmp2*^−/−^ in the neonates generated from the mating of the *Mmp2*^+/−^ females and males demonstrated the following averaged ratios of all the examined litters, as expected according to the Mendelian inheritance: 26.6%, 47.7% and 25.7% for WT, *Mmp2*^+/−^ and *Mmp2*^−/−^, respectively (Figure 1F). Moreover, when the *Mmp2*^+/−^*Mmp9*^+/−^ pairs were mated, and the relative distribution of each genotype was measured at P0, the percentage of the dKO offspring was not lower than expected (Figure S1B). Together, these results rule out any association between fetal death and dystocia in the *Mmp2*^−/−^, *Mmp2*^−/−^*Mmp9*^+/−^ and dKO genotypes.

### 2.2. Mmp2^−/−^ Nulliparous Females Present Relatively Short Uterine Horns

**Figure 2:**
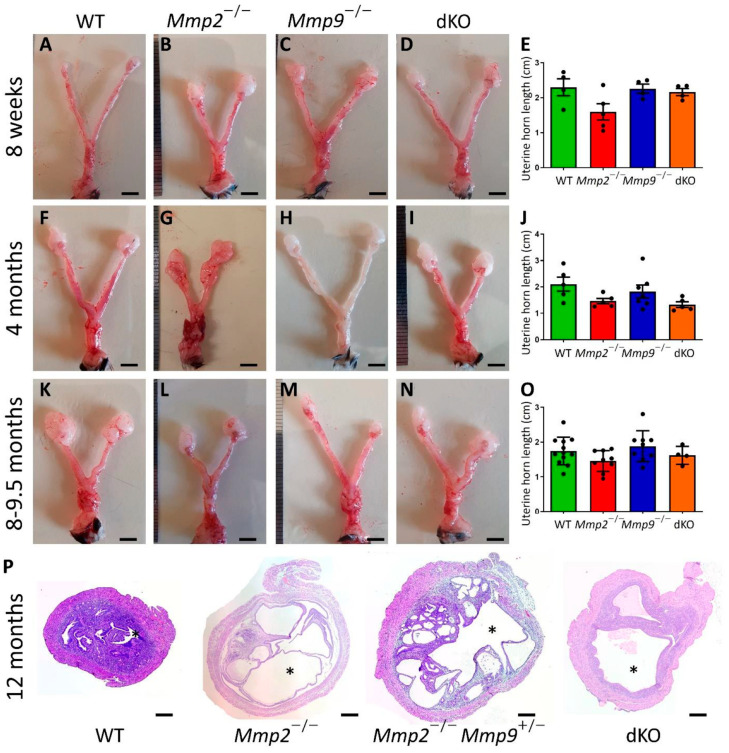
*Mmp2*^−/−^ uteri are shorter in size compared to those of the WT. Representative photographs of 8-week old (**A**–**D**), 4-month old (**F**–**I**) and 8–9.5-month old (**K**–**N**) diestrus-stage uteri collected from all 4 genotypes: WT, *Mmp2*^−/−^, *Mmp9*^−/−^ and dKO. Measurements of the uterine horn length demonstrated that *Mmp2*^−/−^ were shorter at all ages (**E**,**J**,**O**), but not significantly. (**P**) Nulliparous uterine cross-section of *Mmp2*^−/−^, *Mmp2*^−/−^*Mmp9*^+/−^ and dKO, showing significant enlargement of the lumenal area and very thin myometrial layer (compare the lumen indicated by the asterisks). Bar = 4 mm in (**A**–**D**), (**F**–**I**) and (**K**–**N**) and 200 µm in (**P**).

After unraveling the connection between *Mmp2* loss and dystocia occurrence, we next set out to determine the effect of *Mmp2* loss on the uterus morphology. Nulliparous females were used to examine this effect of *Mmp2* loss on the naïve uterine tissue. In mice, the estrus cycle is short (4–5 days) and comprises four precise phases: proestrus, estrus, metestrus and diestrus. As natural morphological changes are known to occur in the uterine wall during the estrus cycle, it is crucial to synchronize the estrus cycle between females, in order to ascertain that morphological differences that may appear in the different genotypes are not estrus-phase related [6,28]. Hence, the females were synchronized and uteri were collected from 4–12 females from the WT, *Mmp2*^−/−^, *Mmp9*^−/−^ and dKO genotypes at three ages: 8 w, 4 M and 8–9.5 M. The decision to examine the females at different ages was based on the observation that dystocia incidents mostly occur among older females (Figure 1D and Figure S1A). The length of the uterus horn was measured and found to be somewhat shorter in the *Mmp2*^−/−^ samples compared to the WT at all the examined ages (Figure 2); however, this difference was not statistically significant, possibly due to the small sample size (*p* = 0.0935, *p* = 0.1991 and *p* = 0.33 for WT vs. *Mmp2*^−/−^ at 8 w, 4 M and 8–9.5 M, respectively). Additionally, the H&E staining of the sections taken from the different uteri at 12 M demonstrated that some *Mmp2*^−/−^, *Mmp2*^−/−^*Mmp9*^+/−^ and dKO samples were markedly abnormal, with extensive luminal areas and secretary glands, compared to the WTs (Figure 2P and Figure S2). These tissues also demonstrated a very thin myometrial layer and almost no endometrium stroma compared to the WTs (Figure 2P). Even though these tissues were collected from unsynchronized females, their abnormalities are most likely not contributed from differences in the estrus phase, since normal uterine tissues in any estrus stage do not present such severe abnormalities [29,30,31].

### 2.3. Mmp2^−/−^ Uterus Demonstrates Enlarged Myometrium, Endometrium and Lumen

**Figure 3:**
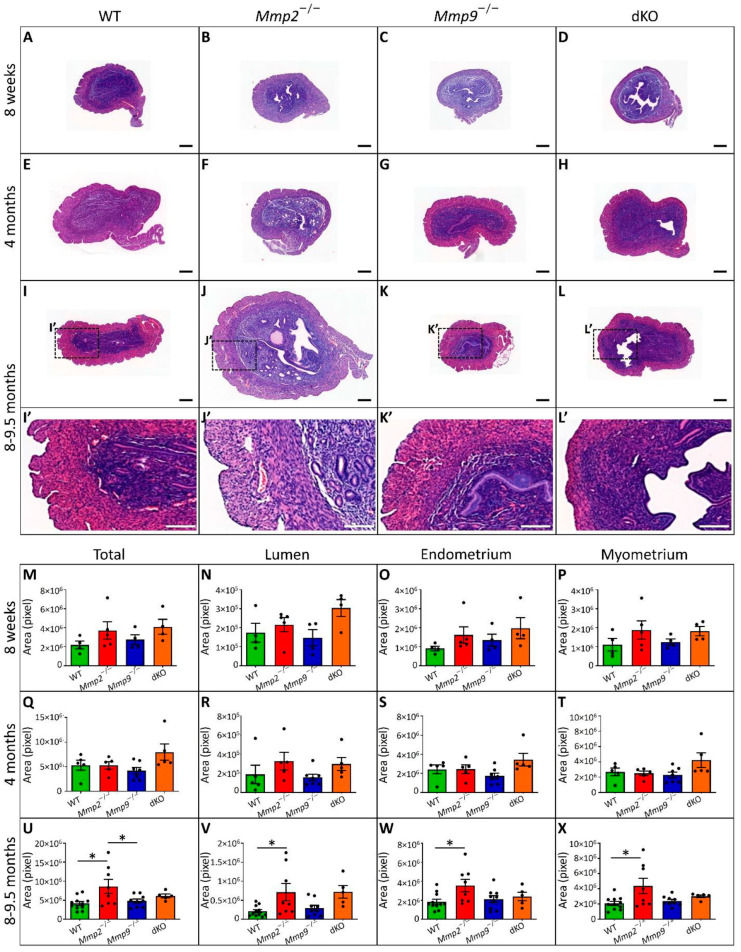
Uterine histology reveals enlargement of the overall uterine tissue in *Mmp2*^−/−^ at 8–9.5 M of age. (**A**–**H**) Histological sections from uterine horn demonstrate small differences in single- or double-KO samples compared to WT, at the ages of 8 w and 4 M. (**I**–**L**) Significant increase in overall tissue size was found at 8 M-old *Mmp2*^−/−^ uteri compared to *Mmp9*^−/−^, dKO and WT. (**I’**–**L’**) Higher magnification of the marked area in (**I**–**L**). (**M**–**T**) Total area and lumen, endometrium and myometrium areas were found to be similar between the different genotypes at the ages of 8 w and 4 M. (**U**–**X**) Specific measurements and statistical analysis show that at 8–9.5 M, *Mmp2*^−/−^ uteri demonstrate a significant enlargement of all measured elements. N = 4–12 for each genotype and age. At (**A**–**L’**), bar = 200 µm. * *p* < 0.05.

To further determine whether the pathophysiology of the dystocia is associated with the abnormal anatomy of the uterus only at older stages, a histological analysis was performed on the uteri harvested at the ages of 8 w, 4 M and 8–9.5 M (Figure 3), similar to those analyzed in Figure 2A–O. We found that at 8 w and 4 M, the uterine tissues of the *Mmp2*^−/−^, *Mmp9*^−/−^ and dKO presented a normal appearance compared to the WT, as determined by the overall tissue size and the relative area measured by each layer of the uterine wall (Figure 3A–H). However, at 4 M, the *Mmp2*^−/−^ tissue started to display a dissimilar structure, with an increased number of secreting glands in the endometrial sub-tissue, compared to other genotypes (Figure 3E–H). However, at the ages of 8–9.5 M, a much more significant enlargement of the entire tissue area was found in the *Mmp2*^−/−^ compared to the other genotypes (Figure 3I–L). Furthermore, specific area measurements of each layer, such as the myometrium, endometrium and lumen, demonstrated that while at 8 w and 4 M, no differences were found between the different genotypes, at 8–9.5 M, the area of all three tissue layers was significantly enlarged in the *Mmp2*^−/−^ compared to the WT (Figure 3U–X, compared to M–P and Q–T).

Notably, concomitantly with the relatively low proportion of the dystocia occurrence in the *Mmp9*^−/−^ females (8.3%, Figure 1), the histology of these uteri did not present major differences compared to the WTs in terms of the total tissue area, or the myometrium, endometrium and lumen areas. Furthermore, the histological characteristics of the dKO (*Mmp2*^−/−^*Mmp9*^−/−^) uteri did not demonstrate a severer phenotype than that of the *Mmp2*^−/−^ at any age (Figure 3). Additionally, the specific measurements of the dKO histology demonstrated that while the total area and the myometrium, endometrium and lumen areas of the *Mmp2*^−/−^ differed from those of the WT significantly (Figure 3U–X), the same parameters in the dKO did not differ from either the *Mmp2*^−/−^ nor the WT. This result, which was in agreement with the less prominent percentage of dystocia observed in the dKO (40%) females compared to the *Mmp2*^−/−^ (54.5%) (Figure 1D), strongly suggests that the additional loss of *Mmp9* against the background of *Mmp2* loss (i.e., in dKO and *Mmp2*^−/−^, respectively) activates a compensatory mechanism that partially resolves the phenotype.

### 2.4. Mmp2^−/−^ Nulliparous Uterus at 8 M Demonstrates Signs of Myometrial Fibrosis

**Figure 4:**
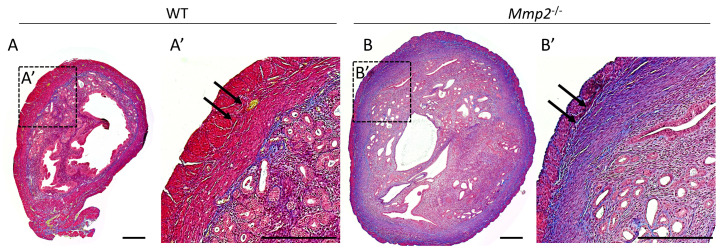
*Mmp2*^−/−^ nulliparous females demonstrate accumulation of collagen fibrils in their uterus. (**A**,**B**) Transverse sections from 8M-old WT and *Mmp2*^−/−^ nulliparous uteri stained with Masson’s trichrome to recognize collagen fibrils (blue, arrows). (**A’**,**B’**) Magnified area in **A** and **B**. Bar = 200 µm.

Collagen accumulation in associated with fibrosis in several tissues, including the liver, kidney and uterus [32,33,34,35,36]. For example, Xu et al. showed enhanced collagen expression and fibrosis in the mouse uterus, which are associated with Notch1 signaling [35]. Furthermore, one of the main substrates known to be degraded by MMP2 is collagen [37,38] and, previously, it was demonstrated that, in cows, there is an increase in MMP2 levels 5 days before calving, which is accompanied with denatured collagen, suggesting that this step enables cervix ripening [26]. Hence, we next asked whether the observed pathophysiology in the *Mmp2*-nulls is coupled with abnormal collagen deposition in the uterine wall, which may be associated with perturbed uterine-wall morphology, as shown in Figure 3. Masson’s trichrome staining for collagen fibrils was performed on transverse sections from the WT and *Mmp2*^−/−^ nulliparous uteri at 8 M, demonstrating increased collagen fibrils in the *Mmp2*^−/−^ tissue, as shown by the enhanced staining (blue), mostly in the circular myometrium, compared to the WT (Figure 4A,B; blue, arrows). Additionally, the Masson trichrome staining on the cervix samples also showed increased levels of collagen in the *Mmp2*^−/−^ compared to the WT (Figure S3A,B). This result suggests a possible mechanism in which the disrupted uterine functionality during parturition in *Mmp2*-KO females is the insufficient remodeling of the uterine ECM, which results in fibrotic myometrium. This fibrosis can be the cause of inadequate contractions of the uterus during parturition, which, in turn, may lead to dystocia. The fact that the other genotypes showed much less accumulation of collagen in the uterine tissue is in agreement with the lower dystocia rates and the relatively normal uterine histology (Figure S3C–F), further highlighting the dominant role of MMP2, rather than MMP9, in this process.

### 2.5. Morphometric Analysis of the Pelvic Bone in Mmp2^−/−^ Females

**Figure 5:**
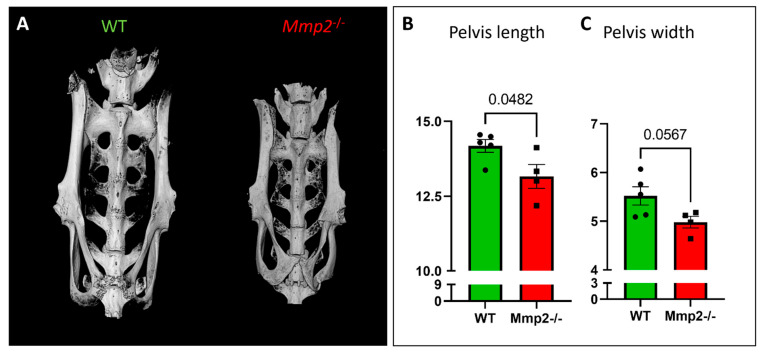
*Mmp2*^−/−^ nulliparous females demonstrate smaller pelvic bones. (**A**) 3D morphometric representation of pelvic bones of WT and *Mmp2*^−/−^ 8 M old females. (**B**,**C**) Measurements of the pelvic bones’ length and width showed significantly shorter pelvises and a tendency towards a smaller space in the pelvises of *Mmp2*^−/−^ females (n = 5 and 4 for WT and *Mmp2*^−/−^, respectively).

Cephalopelvic disproportion (CPD) is a leading cause of dystocia in women, which occurs due to a mismatch between the fetal head size and size of the maternal pelvis, resulting in mechanical “failure to progress” in labor; if untreated, the consequence is obstructed labor, which can endanger the lives of both mother and fetus [39]. Since we previously revealed that *Mmp2* has a role in intramembranous ossification and its loss leads to wider skulls as early as in P0 [23], we next set out to determine whether the pelvic structure is also affected by *Mmp2* loss. Using a µCT scanner, the pelvic bones from the WT and *Mmp2*^−/−^ females at 8 M of age (n = 5 and 4, respectively) were analyzed. Measuring the pelvic bone length and width revealed a significantly shorter pelvic bone in the *Mmp2*^−/−^ females compared to the WT (Figure 5A–C). The pelvic width also demonstrated a great tendency towards being smaller (*p* value = 0.0567), possibly due to the small sample size. Notably, in addition to a smaller pelvis, obstruction can also be associated with fetal overgrowth [40]. However, when measuring the body weights of the P0 newborns, we did not reveal a major weight difference between the *Mmp2*^−/−^ and WT mice, indicating that the dystocia phenomenon does not result from overweight fetuses (Figure S4A). Consequently, the *Mmp2*^−/−^ females of different ages (4 w, 8 w, 6 M) also did not reveal any significant weight differences compared to the WT (Figure S4B–D), suggesting that maternal overweight/underweight is also not associated with the occurrence of dystocia upon *Mmp2*-KO. Altogether, these results raise the possibility of a mechanical mechanism that is involved in the high rates of dystocia in *Mmp2*^−/−^ females, which is driven by the combination of a relatively small pelvic bone with a wider skull in fetuses.

## 3. Discussion

In this study, we examined the effect of *Mmp2* knockout on nulliparous uterine development and its function during parturition process in female mice. We showed that while the KO of the other gelatinase, *Mmp9*, was found to affect parturition only partially, *Mmp2* loss led to a severe defective parturition process with very high rates of dystocia. We demonstrated that 54.5% of all the *Mmp2*^−/−^ pregnancies in the females older than 6 M resulted in dystocia, in which the females presented with at least one dead fetus still in the uterus. The dystocia rate in the WT females was found to be 0% and in the *Mmp9*^−/−^ females, it was less pronounced (15.6%). Surprisingly, the total dystocia proportion decreased in the *Mmp2*^−/−^*Mmp9*^+/−^ and dKO females to 42.9% and 40%, respectively. Furthermore, the histological analysis of the nulliparous uteri of all four genotypes at several ages revealed a significantly enlarged tissue in the *Mmp2*^−/−^, rather than in the *Mmp9*^−/−^, dKO or WT, with increased myometrium, endometrium and lumen areas.

Importantly, a greater accumulation of collagen fibrils was found in the *Mmp2*^−/−^ uterine tissue compared to the WT. As MMP2 is largely known for the proteolytic activity through which it degrades collagen (among other ECM molecules) [38,41,42], it is reasonable to speculate that when this activity is lost, collagen will accumulate in the uterine ECM, leading to myometrial fibrosis. Our data support this possibility and suggest that the increased fibrosis in nulliparous *Mmp2*^−/−^ uteri may be one of the causes of insufficient contractions during parturition, ending with dystocia and in utero fetal death. Moreover, normal levels of collagen fibrils were also demonstrated in the *Mmp9*^−/−^ and dKO uteri (Figure S3), which is in alignment with our observation of lower rates of dystocia in these genotypes. Nevertheless, the dystocia rates that were found in the *Mmp9*^−/−^ (15.6%) are still considered high and non-negligible compared to the WT (0%). This implies that increased collagen deposition, which leads to fibrosis, is most likely not the only mechanism involved.

Furthermore, while, during pregnancy, the cervix must be firm to prevent premature fetus expulsion, when reaching term, cervical ripening is initiated by a cascade of events that includes ECM remodeling, making the cervix more compliant with parturition [3,43,44]. Based on the higher accumulation of collagen fibrils, both in the uterine myometrium and in the cervixes of *Mmp2*^−/−^ females, it is possible that, in addition to the uteri, the cervixes of these females also fail to properly remodel the ECM, resulting in cervical fibrosis. Our suggested role for MMP2 in uterus-ECM remodeling is further supported by previous studies on mice which were knocked out for Anthrax toxin receptor 2 (*Antxr2*) and presented defective parturition and dystocia [45,46]. It was suggested that this resulted from fibrosis in the uterus and cervix due to the aberrant deposition of collagens and fibronectin, together with disrupted myometrial cell layers. Notably, a decrease in the active form of MMP2 was found in the *Antxr2*^−/−^ uteri, leading to the hypothesis that ANTXR2 activates MMP2 by regulating MMP14 activity in the uterus. Furthermore, a different study found in Geranylgeranyl pyrophosphate synthase (*Ggps1)*-KO mice that 75% of all pregnancies ended with dystocia [47]. The isolation of uterine muscle strips from nonpregnant mice revealed that the spontaneous contraction rate and amplitude in the *Ggps1*-KO mice was largely decreased. Additionally, the activation of the Rho/Rho-associated protein kinase (Rock) pathway, which is associated with smooth-muscle contraction, was significantly decreased in the myometria of *Ggps1*-nulls. Thus, the authors concluded that *Ggps1* deletion disrupts the RhoA/Rock2 pathway, causing uterine contraction and parturition problems. Interestingly, Rho was previously found to regulate MMP expression in various cell types [48,49,50], raising the possibility that the Rho/Rock2 pathway is also involved in governing parturition processes in *Mmp2*^−/−^ females. Moreover, it is well known that both MMP2 and MMP9 participate in the remodeling of the mammary gland during pregnancy, lactation and involution [51,52]. Additionally, it was found that *Mmp2*^−/−^ mice show differences in mammary-gland structure and impaired lactation [53]. Therefore, it will be of great interest to investigate in the future whether the loss of *Mmp2* also perturbs tissue remodeling in other gestation-related organs, such as the mammary gland and ovaries.

Dystocia in women is often associated with cephalopelvic disproportion (CPD), which arises when the fetal head size does not align with the size of the maternal pelvis [39]. This mismatch leads to a mechanical “failure to progress” during labor. If left untreated, this can result in obstructed labor, posing a significant risk to the lives of both the mother and the fetus [39]. Therefore, an additional possible mechanism for the dystocia in *Mmp2*^−/−^ mice may be related to skeletal pathology [23]. Indeed, we recently uncovered that during skeleton development, MMP2 participates in intramembranous ossification and its loss results in shorter but wider skulls in neonates [23]. Since the pelvic bones are also composed of irregular and flat bones, which develop through intramembranous ossification [54,55,56], we raised the hypothesis that the pelvic bones of the *Mmp2*^−/−^ females may have been impaired. The measurement of the lengths and widths of the pelvic bones demonstrated a clear trend towards smaller pelvic bones in *Mmp2*^−/−^ females compared to the WT, leading to thinner spaces in the birth canals. Based on this result, we cannot rule out that, similarly to the skull, the pelvic bones of the *Mmp2*^−/−^ mice developed abnormally. Thus, the impairment of the pelvis in *Mmp2*^−/−^ pregnant females, along with the wider skulls we observed previously in the *Mmp2*^−/−^ fetuses, can lead to CPD and consequent dystocia, as previously reported in women [39]. Moreover, the fact that some, but not all, of the P0 *Mmp2*^−/−^ newborns had significantly wider skulls [23], can explain why some fetuses are expelled from the uterus while others become stuck and die.

Interestingly, past analyses of the different phenotypes of various *Mmp*-knockout mice, such as *Mmp1*, *Mmp2*, *Mmp3*, *Mmp7*, *Mmp9*, *Mmp11*, *Mmp12* and *Mmp14*, along with Timp1, Timp2 and Timp3 Kos, did not report an impact on the reproductive axis [57,58,59]. However, subsequent analyses revealed effects on reproduction, such as lower pregnancy rates in Timp1-null females compared to WTs (52% vs. 78%), as well as significantly fewer pups per litter [25,60]. Since it was found, in fibrosarcoma cells in vitro, that TIMP is able to activate proMMP2 by binding its hemopexin domain [59], further studies are needed to explore whether MMP2 activity is impaired in *Timp1*-null uteri.

A confounding factor when aiming to disclose MMPs’ action in any physiological or pathological condition is that the removal of one MMP often results in compensation by other MMP sub-members. For example, the deletion of *Mmp7* results in a 10–12-fold increase in *Mmp3* and *Mmp10* during uterine involution [61]. Furthermore, our previous studies highlighted that the loss of one gelatinase (*Mmp2* or *Mmp9*) in embryonic NCCs is compensated by the other, leading to the normal migration of NCCs [21]. Concomitantly, our recent RNAseq analysis on bone tissue revealed that the loss of both gelatinases leads to an increase in the expression of other MMPs, such as *Mmp13*, *Mmp14* and *Mmp15* [23]. However, this was not evident in the single KOs (each displaying a different bone phenotype), indicating that while the activity of both gelatinases is not redundant in the different bones, only in the absence of both MMP2 and MMP9, the transcription levels of other MMPs become elevated. Based on our findings, it is also possible that in the reproductive system, the genetic loss of one MMP may be compensated by other MMPs, preventing the occurrence of a specific fertility-related phenotype in animal models. Thus, it can be suggested that the different results observed in the *Mmp2*^−/−^ and *Mmp9*^−/−^ females occur through a unilateral compensation mechanism between the two gelatinases, through which *Mmp2* can compensate, to some degree, for the loss of *Mmp9*, but not vice versa; hence, the remaining *Mmp9* alleles in *Mmp2*^−/−^ cannot rescue the defective parturition phenotype. Moreover, as MMPs are secreted as pro-proteins, which must be cleaved in order to become active, the compensation by other MMPs may relate to their enzymatic activation/inhibition, in addition to their expression levels. Therefore, a multiomics analysis [62,63] is required to compare between the transcriptome, proteome and degradome profiles of the uterine tissues in the different genotypes to elucidate the full spectrum of MMPs and other substrates involved in uterine-wall remodeling in the presence or absence of MMP2 and MMP9.

## 4. Conclusions, Strengths and Limitations

This study demonstrates that more than 54% of *Mmp2*^−/−^ pregnancies in females over the age of 6 M end with severe dystocia. This percentage is reduced to 42.9% and 40% with the additional loss of one or both alleles of the other gelatinase, *Mmp9*, respectively, i.e., in *Mmp2*^−/−^*Mmp9*^+/−^ and dKO. The histological analysis revealed increased and abnormal uterine-tissue sizes and suggested a mechanism through which *Mmp2* loss leads to collagen accumulation and fibrosis in the myometrium, preventing it from contracting efficiently. Additionally, a smaller pelvis was demonstrated in the *Mmp2*^−/−^ compared to the WT females, suggesting the contribution of a biomechanical factor to the high rates of dystocia in *Mmp2*^−/−^ females.

The main strength of this study is that this was the first time *Mmp2*/9 loss was studied in the context of physiological parturition processes in mice. The extremely high rates of dystocia found in the *Mmp2*-nulls strongly suggest that this gelatinase is a fundamental regulator of uterine functionality during parturition. The causal relationship between the severe dystocia and the abnormal structure of the uterine wall in the *Mmp2*-nulls shown in this study calls for a further in-depth analysis of the action of gelatinases during normal and abnormal labor, and of the compensatory mechanisms that may be present or missing in the absence of one or both gelatinases. However, while mice are the gold standard for biomedical research, the conclusions from our genetically modified mouse models cannot be directly applied to obstructive or pre-term births in women. Hence, further studies are required to illuminate the roles and mechanisms of action of gelatinases in dystocia etiology and prevention.

## 5. Materials and Methods

### 5.1. Animals

We used *Mmp2*^−/−^ [64,65], *Mmp9*^−/−^ [57], dKO (*Mmp2*^−/−^*Mmp9*^−/−^) [23] and wild-type (WT) C57BL/6J female mice. The WT and *Mmp9*^−/−^ mice were purchased from Harlan laboratories (Rehovot, Israel), while *Mmp2*^−/−^ mice were provided by Martignetti Lab (Mount Sinai School of Medicine, NY, USA). The dKO mice, as well as additional genotypes (*Mmp2*^+/−^, *Mmp9*^+/−^, *Mmp2*^+/−^*Mmp9*^+/−^, *Mmp2*^−/−^*Mmp9*^+/−^, *Mmp2*^+/−^*Mmp9*^−/−^), were generated by us [21,23]. Mice were maintained at the Hebrew University Specific Pathogen Free animal facility according to animal-care regulations. All procedures were approved by the Hebrew University Animal Care Committee (license 21-16781-3). Genotyping was performed as previously described [21].

### 5.2. Dystocia Assessment

Mating in the study was conducted as part of breeding-colony maintenance for other studies [21,23]. Females of different genotypes were mated with males of different genotypes. Dystocia percentages were measured for each female’s genotype as the number of pregnancies ending in dystocia versus the total number of pregnancies for the same genotype.

Dystocia was diagnosed in three different situations: (1) (most common) when a pregnancy reached GD22.5 according to vaginal plug observation, but did not result in normal parturition, as reflected by females that still had one or more (dead) fetuses in their uterus; (2) vaginal bleeding at GD18.5–GD21.5 with remaining of dead fetuses in uterus, which could not be expelled from the uterus even on the next day; (3) when GD was not determined by vaginal plug, but it was clear upon visual observation that females had reached term without apparent parturition, or newborns in the cage but with dead fetuses remaining in uterus and deterioration in females’ physical condition (hunched posture). Notably, in all of the above cases, females presented with reduced muscle tone in their abdomen, which was observed by elevation of the uterus towards the mouse’s chest (as shown in Figure 1A).

### 5.3. Genotype Distribution and Litter-Size Measurements

Males and females from *Mmp2*^+/−^ genotype (n = 4) were mated at the ages of 4–8 months overnight (~16 h), and then females were monitored for vaginal plug, which was determined as gestational day (GD) 0.5. Eighteen days later, at GD18.5, females were euthanized, and genotyping was performed on a piece of tail from their fetuses. The relative distribution of each possible genotype (WT, *Mmp2*^+/−^ and *Mmp2*^−/−^) was calculated and compared to the expected ratio according to Mendel’s law for genetic distribution.

For litter-size measurements, 37 pairs of WT females and males were mated, and the number of neonates was counted at postnatal day (P) 0. The same procedure was conducted for 11 pairs of *Mmp2*^−/−^, 11 pairs of *Mmp9*^−/−^ and 11 pairs of dKO females and males.

### 5.4. Collection of Uterine Tissues

Nulliparous females were euthanized at the ages of 8 weeks (w), 4 months (M) and 8–9.5 M (n = 4–12) to collect their uterine tissues. Prior to that, females were treated with Depo-Provera (Pfizer Inc., New York City, NY, USA), which contains medroxyprogesterone acetate (MPA), for synchronization into diestrus stage. Diestrus stage was chosen according to our previous studies and experience [66]. The MPA (3 mg per mouse) was subcutaneously injected once on day 1 and a second time on day 5. Synchronized diestrus stage was reached 7 days after first Depo-Provera administration [67]. When indicated, uteri at 12 M of age were collected without estrus-cycle synchronization. After uterine collection, their gross morphology was assessed, followed by histological analysis.

### 5.5. Histology

Uterine tissue was fixed with 4% paraformaldehyde overnight at 4 °C, dehydrated with increased concentrations of ethanol, cleared with xylene and embedded in paraffin. Transverse sections (5 µm) were prepared with microtome (Leica, Germany). For staining, slides were washed twice in xylene for paraffin removal and rehydrated using decreasing ethanol concentrations. The H&E staining and Masson’s trichrome staining were conducted as described previously [68,69,70].

Sections were imaged by light microscopy (Axio Imager M1 with AxioCam MRm camera, Zeiss, Germany) or scanned at ×20 magnification, using a Panoramic Flash III 250 scanner (Dhistech3, The Technion, Haifa, Israel).

### 5.6. µCT Scanning and Morphometric Analysis

Pelvic bones from WT and *Mmp2*^−/−^ females (n = 5 and 4, respectively) were dissected at the ages of 7–11 months. Scanning was performed using a Skyscan 1272 X-ray computed microtomography device. Images were obtained by 100 kV X-ray tube voltages, using a 0.25 mm aluminum filter, at 4000 ms of exposure time, at the highest special resolution of 15 µm. For each specimen, a series of 900 projection images were obtained with a rotation step of 0.5°, averaging two frames, for a total rotation of 180°. Flat-field correction was performed at the beginning of every scan for a specific zone and image format. A stack of 2D X-ray shadow projections was reconstructed to obtain images using NRecon software (version number 1.1.18, 1272 control software; Skyscan, Bruker, Belgium). Reconstructed scans were volume-rendered (Amira software v.6.4, FEI, Hillsboro, OR, USA) to visualize the 3D morphologies of the selected samples [71], to allow measurements of the pelvis’ lengths and widths.

### 5.7. Quantification and Statistical Analysis

All data are expressed as the mean ± SEM (standard error). The significance of differences between groups was determined using JMP 15.0 Statistical Discovery Software (SAS Institute 2000) and GraphPad Prism 9.0 using one-way ANOVA followed by Tukey–Kramer HSD test and *t*-test. Differences were considered significant at *p* ≤ 0.05.

## Data Availability

Data are contained within the article and Supplementary Materials.

## Supplementary Materials

The supporting information can be downloaded at: https://www.mdpi.com/article/10.3390/ijms242316822/s1.
