# Supplementary material for: Mmp2 Deficiency Leads to Defective Parturition and High Dystocia Rates in Mice
Source: Int J Mol Sci. 2023 Nov 27;24(23):16822. doi: 10.3390/ijms242316822 (PMC10706207; doi:10.3390/ijms242316822)
Supplement: Supplementary file 1 [file ijms-24-16822-s001.zip › ijms-2668127-supplementary.pdf]

Mmp2 deficiency leads to defective parturition and high dystocia rates in mice

Supplemental data

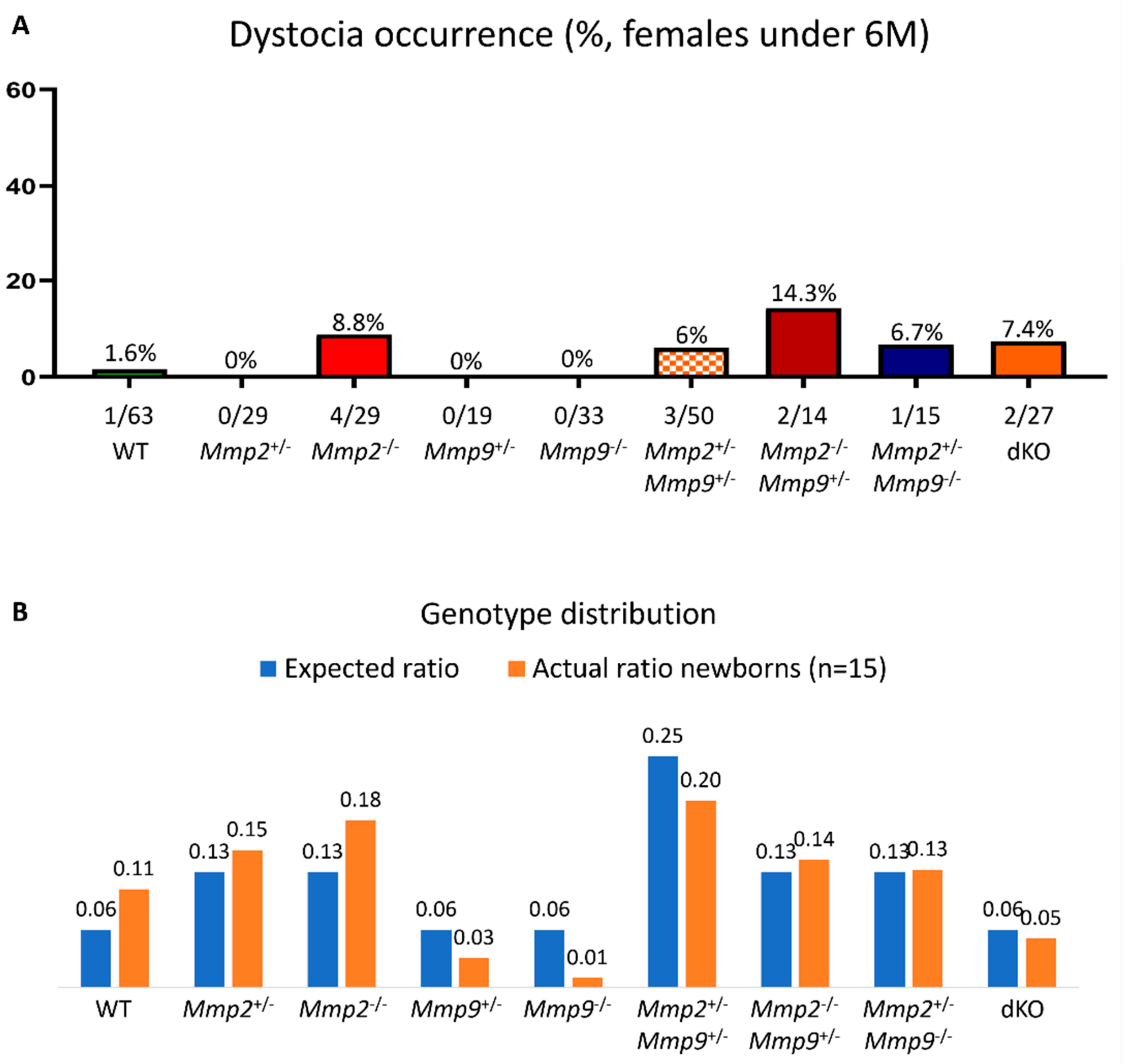

Figure S1. Dystocia occurrence in females younger than 6M and genotypic distribution. (A) Dystocia percentages in females younger than 6 months old. (B) Genotype distribution of newborns born to *Mmp2*$^{+/-}$*Mmp9*$^{+/-}$ males and females (orange) compared to expected ratio according to Mendelian law (blue), n=15 litters.

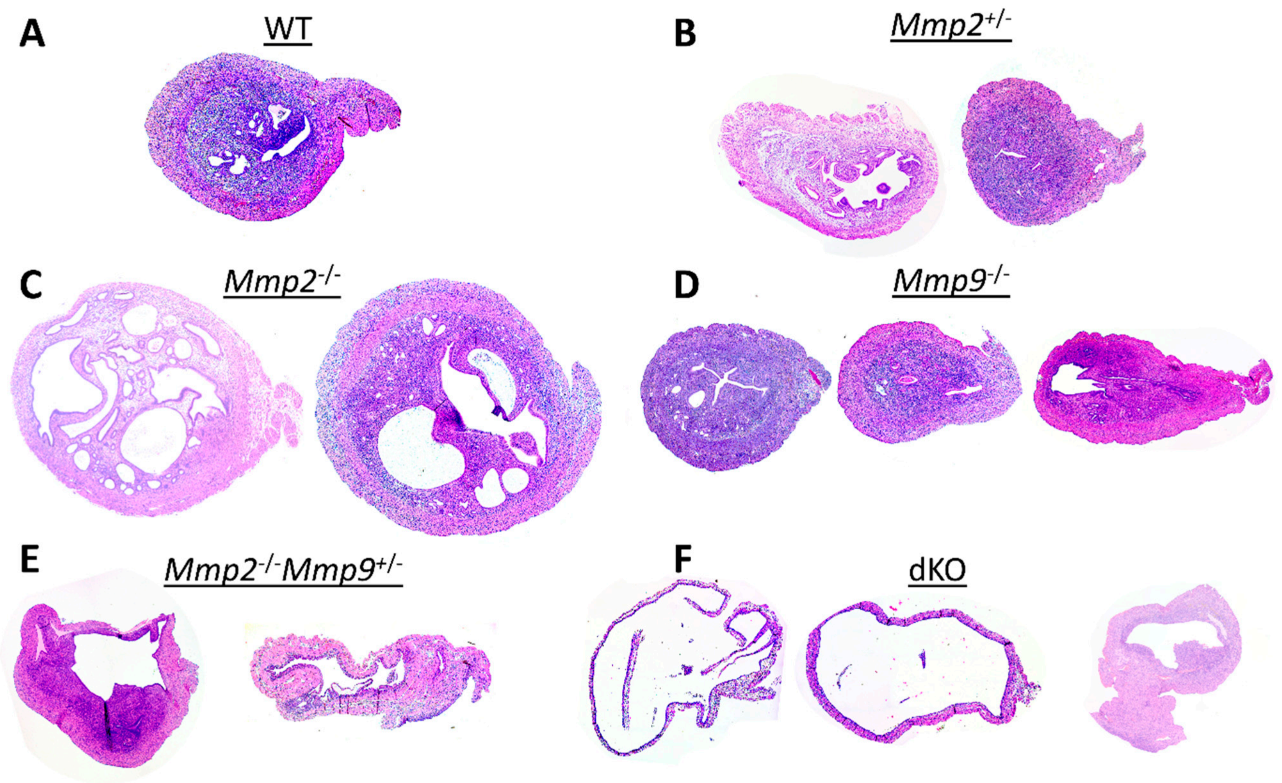

Figure S2. Nulliparous uterine histology at 12M. Uterine histology in *Mmp2*$^{+/-}$ and *Mmp9*$^{-/-}$ is normal compared to WT (compare B and D to A, respectively); but significantly abnormal in *Mmp2*$^{-/-}$, *Mmp2*$^{-/-}$*Mmp9*$^{+/-}$ and dKO compared to WT (compare C, E and F to A, respectively).

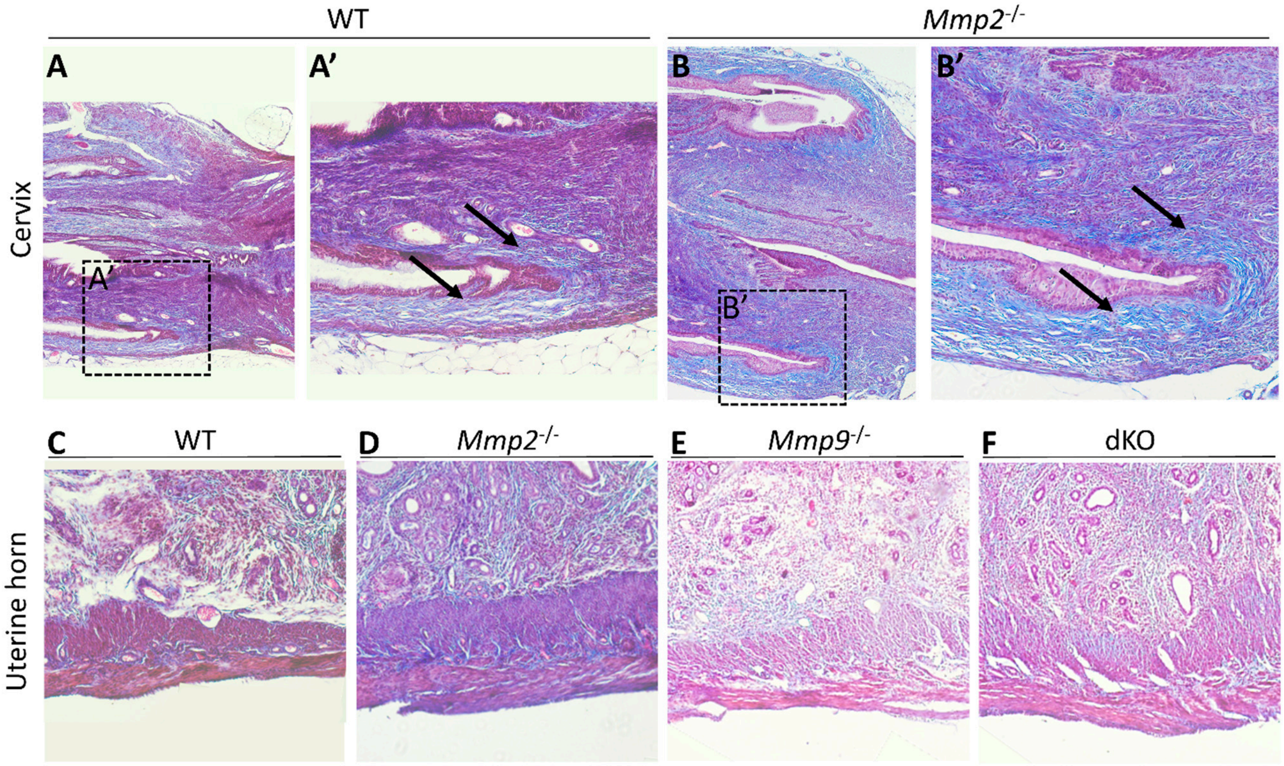

Figure S3. ***Mmp2***$^{-/-}$ **nulliparous females show accumulation of collagen fibrils in their uterine and cervix compared to WT.** (A-B) Sagittal section in the cervix of *Mmp2*$^{-/-}$ (B-B') females show accumulation of collagen fibrils compared to WT (A-A'). (C-F) Transverse sections of uteri from *Mmp9*$^{-/-}$ (E) and dKO (F) females show no increase in collagen accumulation, compared to WT (C), in contrast to *Mmp2*$^{-/-}$ (D).

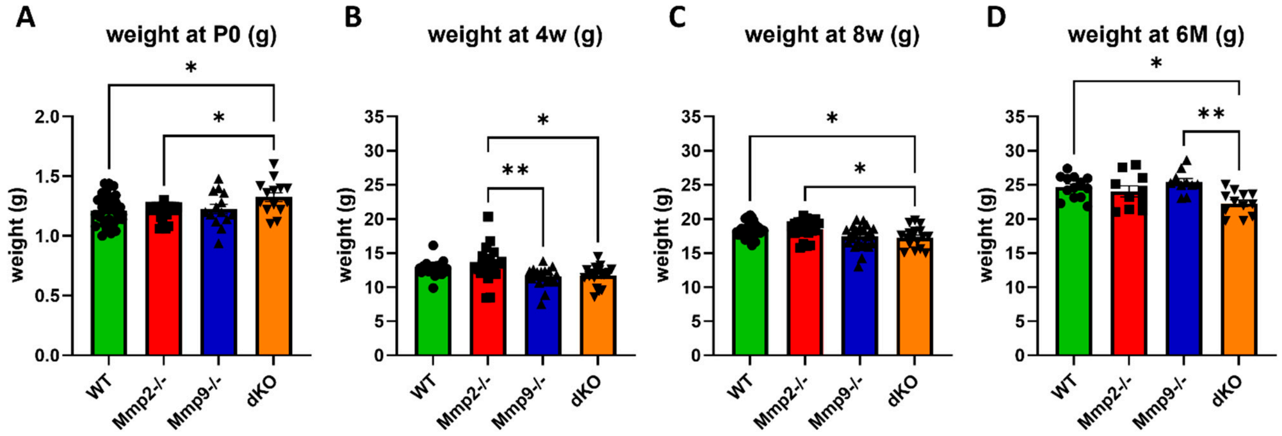

Figure S4. Weight measurements at P0, 4w, 8w, and 6M show not difference between ***Mmp2***$^{-/-}$ **to WT animals.** (A) weight measurements of P0 newborns show that dKO pups weigh significantly more compared to other genotypes. (B-D) weight measurements at 4w, 8w and 6M, respectively, show no difference between Mmp2-/- and WT females. n>10 in each genotype and each measurement.
